# It Protects Them From Me: Reasons Older Adult Black Women Engage in Living-Apart-Together Relationships

**DOI:** 10.1093/geroni/igaa057.1669

**Published:** 2020-12-16

**Authors:** Nytasia Hicks

**Affiliations:** Miami University, Oxford, Ohio, United States

## Abstract

The preference for living-apart-together (LAT) relationships, where individuals are committed to one another but reside in separate households, has increased among older adults. Despite the growing trend to LAT in later life, there is a dearth of literature on living-apart-together exploring the experiences of minority older adult sub-groups. Particularly, few studies have explored motivations for living-apart-together among minority older adult sub-groups. In this study, using a qualitative descriptive approach, reasons older adult Black women engage in living-apart-together relationships were explored. As part of a larger study, thirteen black women ages 59-74 (married and unmarried) completed two semi-structured phone interviews about their motivations for living-apart-together and how decisions, or lack thereof, to LAT were made. Inductive thematic analytic methods revealed three major motivations for LAT among this sub-group. Motivations included: (1) to protect freedom and self-governance; (2) to maintain current living arrangements with live-in family members; and (3) to maximize healthy relationship characteristics (e.g. individuality). Participants reflected that reasons to engage in LAT were influenced by the distance between living-apart-together partners, current caregiving roles, and level of commitment. As to how older adult black women decided to engage in living-apart-together relationships, Participants reported that LAT happened by coincidence or because of a partner’s preference to maintain separate households. Boundary reinforcement regarding role strain was also identified as a core concept. Findings point to the need for applications to variations in relationship types among older adults regarding living arrangements, to professionals supporting aging families, and to further studies of family gerontology.

